# Detection of involved margins in breast specimens with X-ray phase-contrast computed tomography

**DOI:** 10.1038/s41598-021-83330-w

**Published:** 2021-02-11

**Authors:** Lorenzo Massimi, Tamara Suaris, Charlotte K. Hagen, Marco Endrizzi, Peter R. T. Munro, Glafkos Havariyoun, P. M. Sam Hawker, Bennie Smit, Alberto Astolfo, Oliver J. Larkin, Richard M. Waltham, Zoheb Shah, Stephen W. Duffy, Rachel L. Nelan, Anthony Peel, J. Louise Jones, Ian G. Haig, David Bate, Alessandro Olivo

**Affiliations:** 1 Department of Medical Physics and Biomedical Engineering, University College London, Gower St, London, WC1E 6BT UK; 2 St Bartholomew’s Hospital, Barts Health NHS Trust, West Smithfields, London, EC1A 7BE UK; 3 Nikon X-Tek Systems, Tring Business Centre, Icknield Way, Tring, Hertfordshire HP23 4JX UK; 4 Barts and the London School of Medicine and Dentistry, Queen Mary University of London, Newark St, London, E1 2AT UK

**Keywords:** Breast cancer, Cancer imaging, Cancer therapy, Cancer, Optics and photonics, Physics, Applied physics, Optical physics, Techniques and instrumentation, Biomedical engineering

## Abstract

Margins of wide local excisions in breast conserving surgery are tested through histology, which can delay results by days and lead to second operations. Detection of margin involvement intraoperatively would allow the removal of additional tissue during the same intervention. X-ray phase contrast imaging (XPCI) provides soft tissue sensitivity superior to conventional X-rays: we propose its use to detect margin involvement intraoperatively. We have developed a system that can perform phase-based computed tomography (CT) scans in minutes, used it to image 101 specimens approximately half of which contained neoplastic lesions, and compared results against those of a commercial system. Histological analysis was carried out on all specimens and used as the gold standard. XPCI-CT showed higher sensitivity (83%, 95% CI 69–92%) than conventional specimen imaging (32%, 95% CI 20–49%) for detection of lesions at margin, and comparable specificity (83%, 95% CI 70–92% vs 86%, 95% CI 73–93%). Within the limits of this study, in particular that specimens obtained from surplus tissue typically contain small lesions which makes detection more difficult for both methods, we believe it likely that the observed increase in sensitivity will lead to a comparable reduction in the number of re-operations.

## Introduction

Breast-conserving surgery (BCS) followed by adjuvant therapy is the preferred standard of care for early-stage breast cancer^1^. The success of BCS depends on the removal of the entire neoplastic lesion, which is determined through histopathological analysis of the margins of the resected wide local excision (WLE). This is usually only available after several days, when any detected margin involvement must be discussed with the patient and re-operation considered. Re-operations cause patient stress, worse cosmetic outcomes and additional costs to healthcare systems. Their incidence varies among centres, with a recent study showing it can be as high as 41% with a median of 17.2%^2^.

A mechanism to detect margin involvement intraoperatively would allow for the removal of additional tissue during the same intervention thus avoiding re-operations. Most practice (e.g. in the UK) involves the use of planar X-ray radiography^3^, which is affected by the known limitations of X-rays in detecting faint soft-tissue changes^4^, such as those entailed by low-grade ductal carcinoma in situ (DCIS), invasive lobular carcinoma and microscopic margin involvement and tumour strands in general. Other approaches currently in use include intraoperative ultrasonography, which also suffers from similarity in tissue density, and pathology assessment with frozen sections or touch imprint cytology, which are time consuming, require on-site laboratory facilities, and in the latter case only allow localised assessments.

This lack of a satisfactory solution has generated research effort into alternative methods. These include approaches based on optical/infra-red wavelengths such as Raman spectroscopy^5^, Optical Coherence Tomography^6^ and optical sectioning microscopy methods^7,8^, or on radiofrequency^9^; these typically probe specimens only at specific locations, and suffer from limited penetration depth. More recent methods involve administering radionuclides to allow the detection of Cherenkov radiation^10^, which exposes patients and practitioners to radiation, and diathermy smoke detection by mass spectrometry coupled to a surgical scalpel^11^. This shows promise, however it cannot detect close (as opposed to involved) margins, and requires characterisation of molecular signatures for all possible at-risk lesions.

X-ray micro-CT fulfils key requirements of tissue penetration, spatial resolution, simultaneous analysis of the entire specimen in a sufficiently short timeframe, and it has indeed been proposed for intraoperative use^12^, the main obstacle being the limited soft tissue sensitivity of conventional X-ray imaging^4^. This can be overcome by X-ray phase contrast imaging (XPCI), the efficacy of which in the detection of breast tumours has been repeatedly proven^13–16^, including in vivo^17^. This study aims to demonstrate the feasibility and the prospective advantages of using XPCI for intraoperative imaging of WLEs. It should be noted that, since we are comparing our approach to conventional specimen radiology, the enhancements we demonstrate in this paper arise from two factors: 3D visualisation of the specimen, which was already proven to bring some advantages^18–21^, and increased soft-tissue sensitivity arising from XPCI, also proven advantageous by previous studies^13–17^. We focused on the comparison with conventional specimen radiology because it is one of the most commonly used tools in breast conserving surgery; comparisons with more advanced modalities will be the subject of future studies.

## Results

We scanned 101 WLE-like specimens obtained from tissue surplus to central diagnosis with our breadboard XPCI-CT system (see “Methods” section); 99 of these were also scanned with a conventional specimen imaging system. Following phase retrieval and CT reconstruction (see “Methods” section), the team’s radiologist scored all XPCI-CT images for tumour presence; when detected, it was determined whether the lesion reached the specimen’s margins. The same analysis was applied independently to the images obtained with the commercial specimen radiology system. Once the “ground truth” was obtained through histopathology assessment, data were aggregated in the 2 × 2 contingency tables reported below. For all three modalities a lesion distance < 1 mm from the margin was considered as a “positive” margin, in keeping with clinical practice.

Table 1 shows the XPCI-CT results for detection of tumour at margin. Sensitivity and specificity values are 83% (95% CI 69–92%) and 83% (95% CI 70–92%), respectively. Table 2 shows the same results for conventional specimen imaging. Sensitivity and specificity are 32% (95% CI 20–49%) and 86% (95% CI 73–93%), respectively. These results confirm that the main objective of the study has been achieved, namely a statistically significant improvement in sensitivity by XPCI-CT for the detection of involved margins.

**Table 1:** 2 × 2 contingency table for cancer presence at margins as detected by XPCI-CT.

Pathology	XPCI-CT		Total
	Cancer	No cancer	
Cancer	39	8	47
No cancer	9	45	54
Total	48	53	101

**Table 2:** 2 × 2 contingency table for cancer presence at margins detected by specimen radiography.

Pathology	Conventional specimen radiography		Total
	Cancer	No cancer	
Cancer	14	29	43
No cancer	8	48	56
Total	22	77	99

Cases where “tumour at margin” was classed as “unsure” by the radiologist were assigned to the yes category; tables in which uncertain cases were excluded from the analysis are reported in the supplementary information (ST1 and ST2), and show no significant differences in sensitivity and specificity. Tables reporting the detection of tumour regardless of its position in the specimen are also reported for completeness in the supplementary information (ST3 and ST4), again showing no significant differences in the sensitivity and specificity values.

XPCI-CT slices showed a wealth of details invisible in conventional specimen radiography, thanks to the increased sensitivity provided by phase effects combined with the system’s spatial resolution of approximately 100 mm^22^.

**Figure 1:**
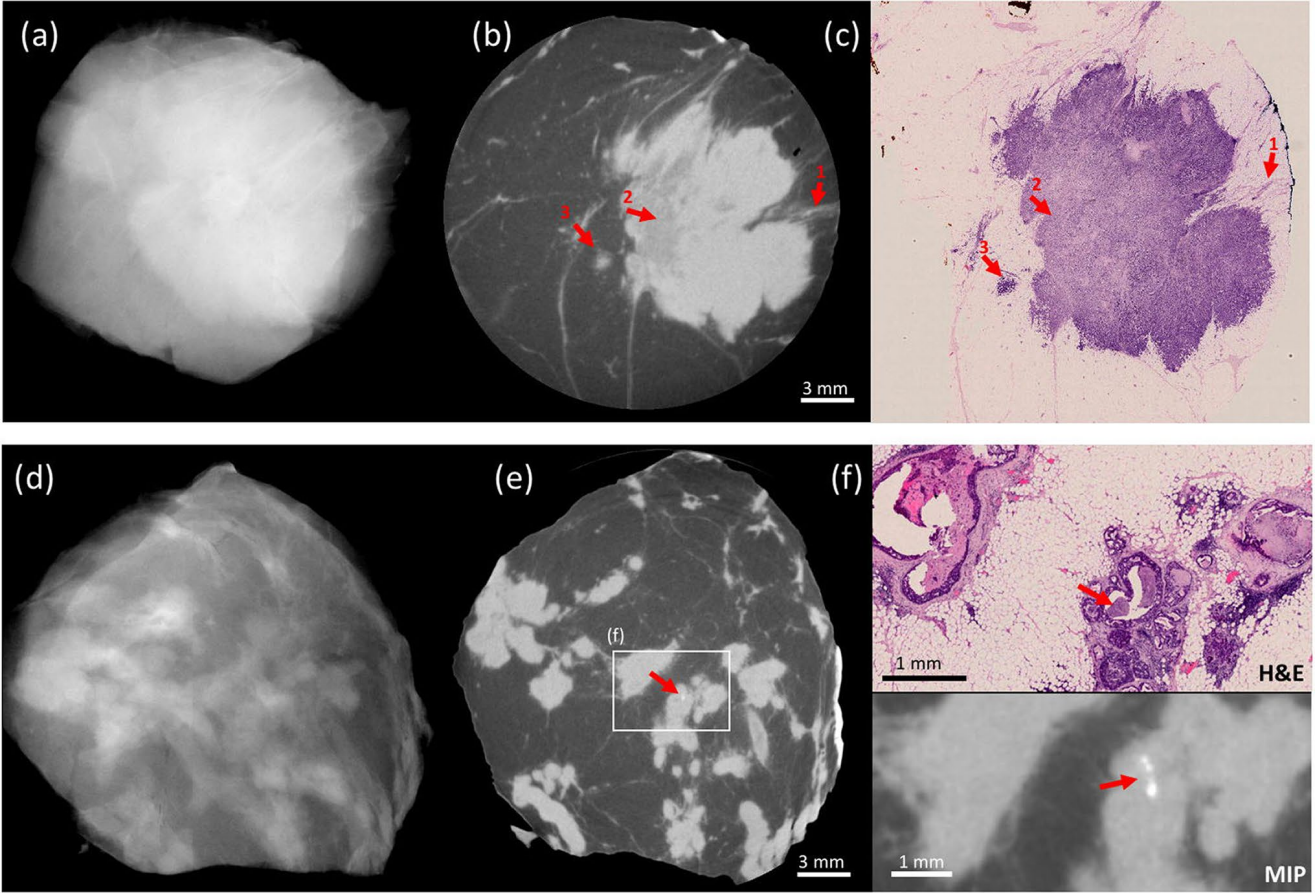
Examples of the imaging performance of XPCI-CT (**b**,**e**) compared to conventional specimen radiography (**a**,**d**) and benchmarked against histopathology (**c**,**f**). The top row focuses on the similarity between the XPCI-CT slice in (**b**) and the histological slice in (**c**). Arrow 1 indicates margin involvement, arrow 2 a variation in density in the internal structure of the tumour mass, arrow 3 tumour-induced inflammation. All this is confirmed by the histological slice in (**c**), and hardly visible in the conventional image in (**a**). The bottom row focuses on the detection of small calcifications, a key feature in DCIS. These are undetectable in (**d**), detected in (**e**), enhanced in the maximum intensity projection (MIP) image at the bottom of (**f**), and confirmed by histopathology in the top part of (**f**). The scale bar [shown in (**b**) and (**e**)] is the same for all images apart from (**f**), which has its own scale. Red arrows in (**e**) and (**f**) indicate the microcalcifications.

Visual comparison between XPCI-CT and histopathological slices reveals a strong similarity between the two. Most details are visible in both modalities, leading to an ease of correlation between corresponding features. Crucially, this includes features that are the main target of this study, including thin tumour strands extending all the way to the margin of the specimen, faint DCIS, small calcifications. Typical examples are reported in Fig. 1, which demonstrates XPCI-CT’s ability to detect faint tumour margins, inhomogeneity inside tumour masses, tumour-induced inflammation and small calcifications in DCIS, all confirmed by histopathology. Similar matches were repeatedly seen across all specimens for which histology and XPCI-CT were compared visually. Many other tumour-related features become visible, not limited to margin assessment. These provide additional information such as multi-focality, variations in tumour structure, 3D pattern of calcifications, necrosis and tumour-induced inflammation.

**Figure 2:**
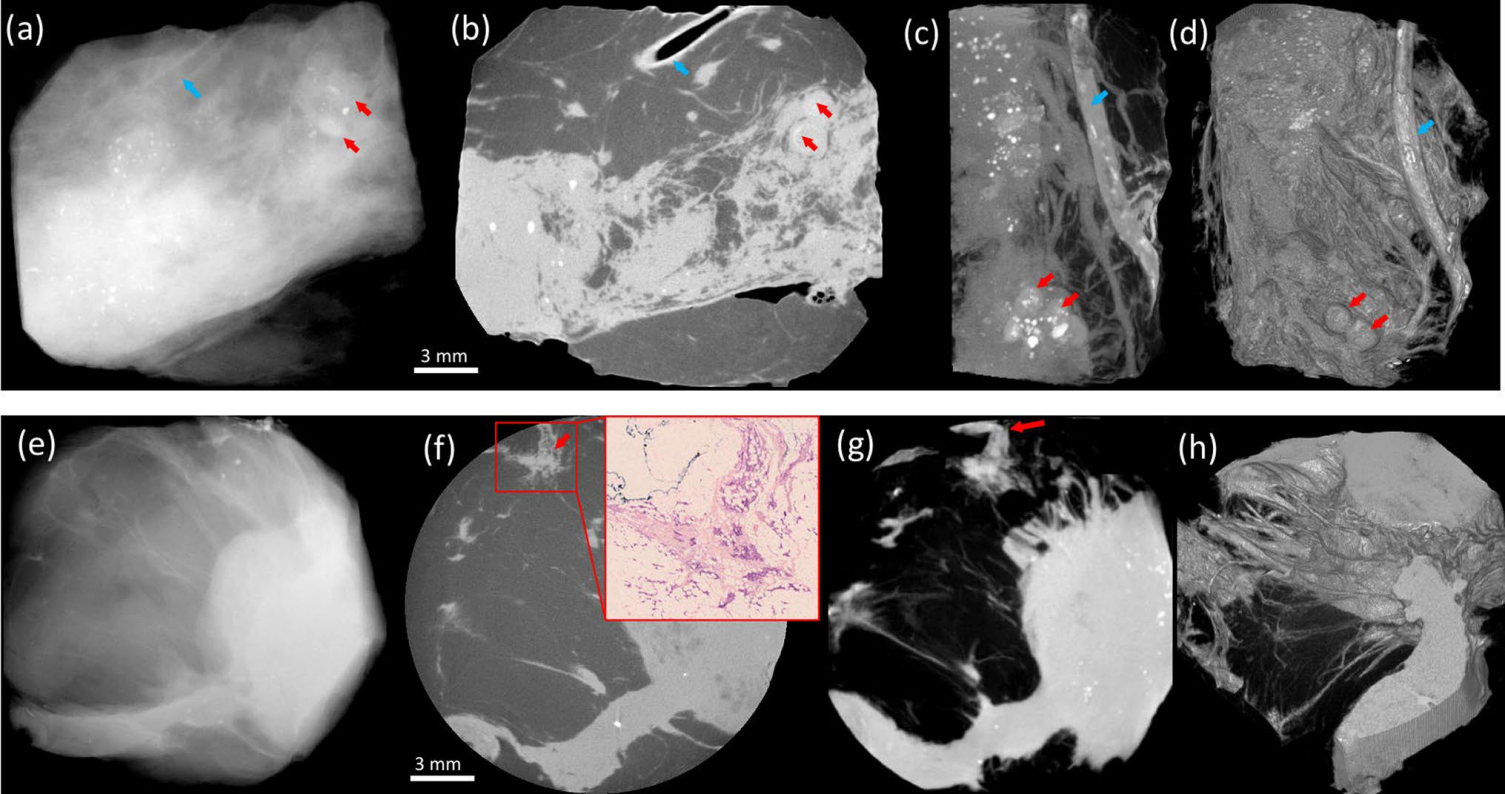
Versatility of representation offered by the XPCI-CT approach. The top row focuses on calcified DCIS, also detectable in the conventional image (**a**). However, the clear delineation of the enlarged ducts in the XPCI slice (**b**) should be noted (red arrows), which can be followed along their length through successive slices. Alternatively, MIPs can be used (**c**), or more sophisticated 3D rendering approaches such as solid casting followed by segmentation (e.g. fat tissue was segmented out in panel d, allowing the visualisation of an entire blood vessel and calcifications therein, blue arrow). The bottom row shows an involved margin, less clearly visible in the conventional image (**e**) and highlighted by an arrow in the XPCI slice (**f**), where the inset shows confirmation by histopathology, and in the MIP in (**g**). The 3D rendering in (**h**) allows visualising edge of the tumour (a metaplastic carcinoma), characterised by diffuse invasion along fibrous strands.

The 3D visualisation means that tumour strands can be identified near the main tumour site where they are thicker, and followed through the volume as they become thinner until they reach the specimen’s margins. This can be done flexibly, e.g. either by following features while scrolling through adjacent slides, through maximum intensity projections or by using 3D rendering representations of the sample, examples of which are provided in Fig. 2. As well as highlighting the improved visualisation of expanded ducts, Fig. 2 provides an additional example of margin invasion by a metaplastic (matrix-producing) carcinoma, again supported by histology confirmation.

### Feasibility of clinical translation

**Figure 3:**
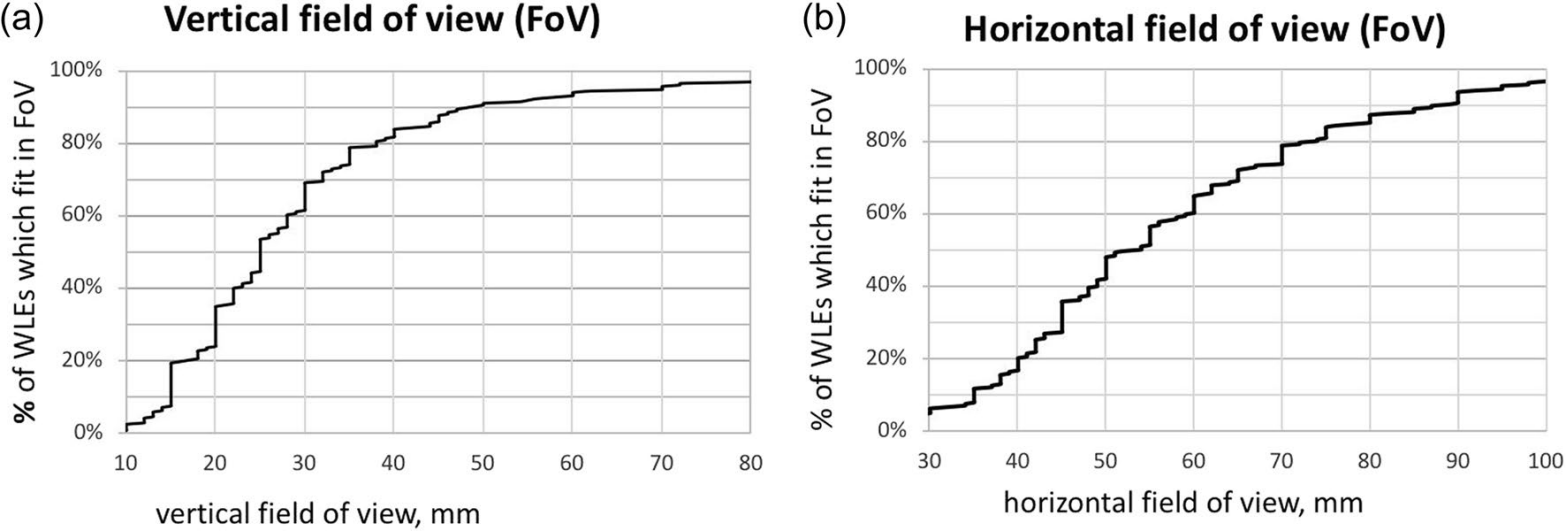
Size distribution of 248 consecutive WLEs examined at London’s Barts Hospital (**a**, vertical; **b**, horizontal).

At the end of the study, encouraged by the positive results, we conducted an investigation into the clinical viability of the proposed technology, in terms of providing a sufficiently large field of view (FoV) and acquisition times sufficiently short for intraoperative use. The first step was to conduct a survey of the average size of 248 WLEs which arrived consecutively at the breast tissue bank of Barts hospital in London (see Fig. 3). This confirmed that a FoV of 9 cm would be sufficient to cover over 90% of the cases.

**Figure 4:**
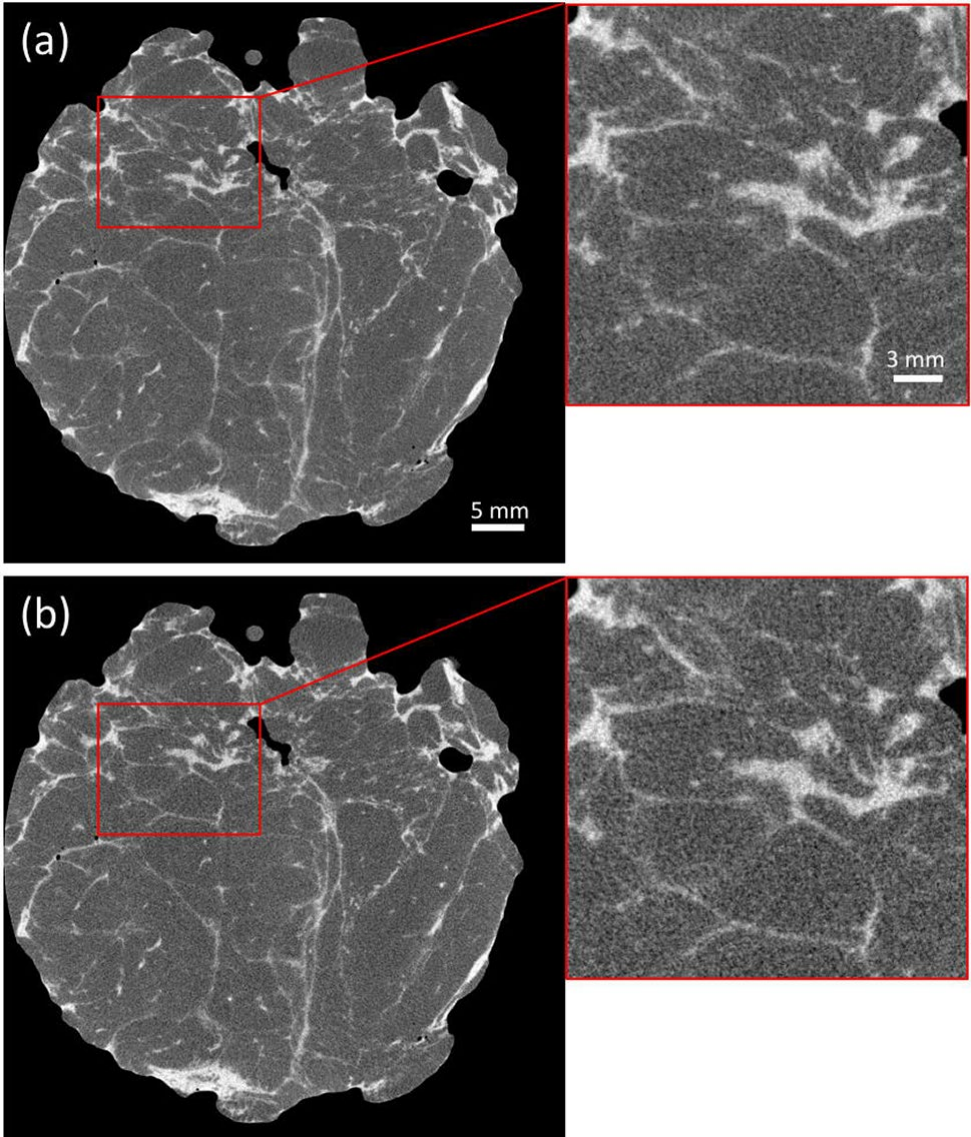
XPCI-CT slices of a 5 cm diameter WLE specimen acquired with an overall scan time of 15 min (**a**) and 10 min (**b**). Zoomed-up regions of interest show images on the same scale as Figs. 1 and 2.

Based on this knowledge, we designed new pre-sample and detector masks analogous to those described in the materials and methods section below, but with overall areas equal to 9 × 9 cm^2^ and 11.5 × 11.5 cm^2^, respectively. Masks were then incorporated into a cabinet-based pre-commercial prototype featuring the same X-ray source and detector as the breadboard system; however, a modification in the detector firmware was requested to the manufacturer (Hamamatsu), in order to enable shorter (0.4 s) acquisition times for the individual frames, thus allowing faster CT acquisitions. The system was tested on a larger (approx. 5 cm diameter), fresh WLE specimen resected immediately before the scan, with results for two different acquisition times (15 and 10 min) shown in Fig. 4.

Albeit a grainier texture, resulting from the combination of faster scans and thicker samples, becomes apparent in the zoomed-up regions of interest, all key image features remain visible, despite the larger specimen size and scan times being reduced to a level compatible with intraoperative use. Notably, image quality is only marginally affected when the scan time is reduced from 15 to 10 min, indicating the possibility for additional reductions, which will be explored in a dedicated study. Image reconstruction took an additional 10–15 min (on a 24-core PC), however this was not optimised yet at this stage.

## Discussion

This study was aimed at demonstrating the feasibility and prospective advantages of using XPCI for intraoperative imaging of WLEs.

Demonstration of prospective advantages was addressed by analysing 101 WLE-like specimens obtained from surplus tissue. These specimens had two key differences with respect to real WLEs: tumour presence was uncertain (and unknown to the investigators until after the results of histological workup were disclosed to the team) and, where tumour was present, this could be small in size since the main lesion had already been removed from the original specimen for diagnostic purposes. This is likely to have made lesions more difficult to detect than in real WLEs, which could explain the limited sensitivity performance of the conventional specimen imaging system. In addition, in normal clinical practice the radiologist would also use information available from the pre-operative imaging, which is not available in this case. However, sensitivities as low as 20% have been reported in the literature for standard specimen mammography^23^, with other studies reporting only marginally higher sensitivity values for certain cancers^24^. Smaller and fainter lesions would be harder to detect for both methods, and our study focused on their relative comparison.

We found comparable specificity, but a significantly enhanced sensitivity for XPCI-CT. A significantly increased sensitivity was the main target of our study, as it is key to achieve a more efficient detection of margin involvement, and therefore a reduction of the reoperation rate. The high similarity between XPCI-CT and histological slices, and the wealth of additional details which can be detected and identified compared to conventional specimen imaging, provides a further, strong indication of the prospective usefulness of XPCI-CT becoming available intraoperatively.

To demonstrate feasibility and compatibility with clinical requirements, we developed a pre-commercial prototype based on the breadboard system design, but with larger masks and modified detector firmware to deliver faster frame rates. This enabled reaching a FoV of 9 × 9 cm^2^, which we demonstrated would be sufficient to cover over 90% of the cases encountered in clinical practice by analysing the size distribution of 248 consecutive WLEs collected at our reference hospital. The pre-commercial prototype also enabled demonstrating an overall scan time of 10 min, the further reduction of which will be investigated in a future study, including across a range of WLE sizes and types of malignancy. In particular, it would be important to provide a breakdown of sensitivity and specificity versus lesion type, for which at the current stage we felt we did not have a sufficient statistic for each case to support statistically significant conclusions.

In summary, the obtained gain in sensitivity shows that clinical introduction of the technology would be beneficial and translate into a comparable reduction in re-operations; the demonstration that this can be attained with a compact system featuring a FoV sufficient to cover over 90% of WLEs at scan times compatible with intraoperative use proves it is viable. It would also be the only fully 3D method available for intra-operative specimen imaging, with a resolution of the order of 100 μm over the entire volume^22^, and soft tissue sensitivity sufficient to detect faint tumour features. It would allow the operator to follow tumour strands from the main mass where they are thicker and easily recognisable, and determine whether they reach the specimen margin.

Once introduced, the technology could provide additional advantages. As well as CT scans in minutes, the XPCI machine can provide planar (projection) images in seconds. Alongside “single shot”^25,26^ images enhancing visibility of tumour features, “multi-modal” 2D images can be obtained, in which the contributions of X-ray attenuation, phase and potentially scatter are separated out, highlighting different features of the specimen (see Fig. S1 in the supplementary information and related discussion). This lends itself to a “hybrid” use where the surgeon or radiologist could rapidly examine the WLE in 2D from different orientations, and launch a full CT scan only when the 2D images are insufficient to confidently determine whether margin involvement is present. The ability to detect metastatic tumour in sentinel lymph nodes also could be explored, providing further potential to reduce second operations due to unexpected positive nodes. Applications can be envisaged beyond breast, to other areas where a 3D method with high soft tissue sensitivity would be beneficial (e.g. intestinal, oesophageal or prostatic surgery). The machine could be used to aid slide selection in pathology, especially for multi-focal lesions, or simply by providing a three-dimensional “digital histology” support. It would also help accelerating the clinical uptake of XPCI, alongside pilots such as the mammography study with synchrotron radiation in Trieste, Italy^17^ and the lung study underway in Munich, Germany^27^.

Our study’s limitations arise from its still preliminary nature, dictated by the need to demonstrate its potential without disrupting the clinical workflow, which imposed the use of surplus tissue. Additional limitations may come from image assessment being conducted by a single multi-disciplinary medical team, which however represents the situation encountered in clinical practice for BCS. Future work needs to include teams from a variety of hospitals. Finally, it must be noted that, despite the enhanced sensitivity, XPCI-CT still misses some cancers ultimately detected by histopathology, hence further improvements should be pursued. It became clear during the study that additional training should be provided to the radiologist; however, encouraging indications were provided by the reasonably high levels of specificity that could be achieved also with minimal training, which we expect leaves room for further improvement.

Our key result is a > 50% increase in sensitivity over conventional specimen radiology (from 32 to 83%). Although the performance of the conventional system is likely to have been affected by the use of WLE-like specimens created using surplus tissue, which resulted in smaller and fainter lesions compared to real WLEs, this would have comparably affected the performance of XPCI-CT. Within the limits of the present preliminary study, this result indicates that introduction of XPCI-CT as an intra-operative specimen imaging tool could lead to a reduction in repeat operations by a rate comparable to the observed increase in sensitivity over the conventional method.

## Methods

### X-ray phase contrast CT

XPCI exploits the changes in speed with which X-rays travel through different tissues, a stronger effect than the variations in X-ray attenuation caused by the same tissues^28^. This was proven to enhance soft tissue contrast, including of breast tumours^13–17^. Several methods have been developed to translate phase effects into image contrast^13–17,28–36^; importantly, implementations with conventional X-ray sources are now possible^31,32^, including in CT^33–36^. Early CT implementations required multiple images at each projection angle to “disentangle” phase from attenuation effects (phase retrieval). This prevented a continuous rotation of the specimen, leading to acquisition times too long for intraoperative use. We developed an adaptation^26^ of a “single-shot” approach^25^ in which a pre-sample mask is kept stationary throughout the scan, and the object is continuously rotated. “Single shot” approaches^25,26^ enable phase retrieval only for specimens which are fairly homogeneous in composition, which works well for BCS since WLEs are mainly soft tissue.

**Figure 5:**
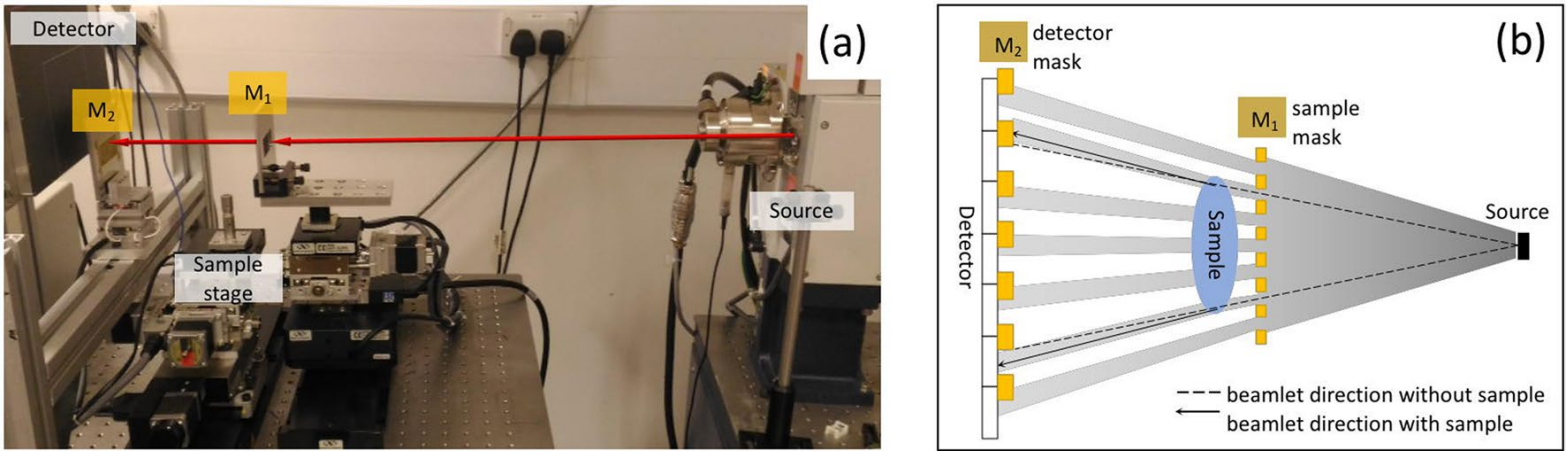
The breadboard XPCI-CT system. (**a**) A photograph of the system, with flat panel detector, the two masks in aluminium frames and the X-ray source (on a separate table) recognisable from left to right. (**b**) A schematic of the system (seen from above, not to scale), showing how mask M1 splits the beam into multiple beamlets before the sample, and how these hit the edges of an aperture on the detector mask M2. The way in which small deviations of the beamlets lead to a change of detected intensity in some pixels is also schematized.

This study used a custom-built bread-board scanner^22,36^, 70 cm long with a 15 cm sample-to-detector distance. A schematic is provided in Fig. 5, alongside a photo of the setup. It features a rotating anode Molybdenum source (Rigaku 007-HF Micro Max, Rigaku, Japan) operated at 40 kVp and 20 mA, a CMOS-based flat panel detector (C9732DK-11, Hamamatsu, Japan) with 50 μm pixel size. The key difference over a conventional micro-CT system is the introduction of two masks featuring long, regularly spaced vertical apertures. One mask (area 5.5 (H) × 2.0 (V) cm^2^, aperture size 12 μm, aperture period 38 μm) is placed immediately before the specimen, the other (area 6.0 (H) × 2.5 (V) cm^2^, aperture size 20 μm, aperture period 48 μm) in front of the detector. A lateral displacement between the masks enhances the signal created by X-rays that deviated from their original direction, i.e. the phase contrast (see Fig. 5b); for this system, a 9 μm displacement results in maximum phase sensitivity^22^. Both masks were fabricated to the authors’ design by Microworks GmbH (Karlsruhe, Germany) by electroplating an approximately 120 μm thick gold layer on a patterned 400 μm thick graphite substrate. 2500 projections were collected while the specimen was rotated continuously over 360 degrees at a speed of 0.1 °/s. This 1 h scan duration was reduced to 10 min in an optimised pre-commercial prototype employing a higher X-ray flux and a detector with a faster frame rate (see “Feasibility of clinical translation” section). This pre-commercial system also employed much larger masks (9 × 9 cm^2^ and 11.5 × 11.5 cm^2^ for sample and detector mask respectively, both still fabricated by Microworks GmbH), to allow scanning larger specimens.

### Specimen preparation, conventional radiography and histopathology

101 WLE-like specimens were obtained from tissue surplus to central diagnosis, to avoid disruptions to the clinical workflow. All samples were obtained from patients who consented for use of their tissue in research as part of the Barts Cancer Institute Breast Tissue Bank (Ref:15/EE/0192). For ease of transportation, storage and scanning, all specimens were fixed in 10% formal saline for at least 24 h. To ensure this would not affect the results of our study, the same specimen was scanned before and after fixation, and no significant difference was observed in the resulting images by the team’s radiologist, or in the extracted quantitative contrast values (see supplementary information, Fig. S2 and Table ST5).

All specimens underwent XPCI-CT; 99 were also imaged post-fixation with the hospital’s specimen radiography system (Bioptics Core Vision, Bioptics Inc, Tucson, USA), to allow a direct comparison with current practice. Following imaging with both systems, histological examination was carried out on all specimens. The entire WLE-like sample was blocked out for analysis, taking full-face slices into conventional or megablock cassettes. These underwent routine processing and embedding in paraffin wax. A 4 μm section was taken from each block and stained with haematoxylin and eosin according to standard protocols. In selected cases, deeper levels were taken through the block to ensure full representation of the lesion.

### Image reading and statistical analysis

The team’s radiologist blindly assessed all images and assigned them to one of three categories: cancer present, cancer at margin or no cancer. While conventional images posed no issue due to their familiar appearance, XPCI-CT images required some degree of training, since their enhanced sensitivity meant many more details became visible compared to what is normally observed in clinical practice (see Fig. 1 as an example). Without training, these additional features could be interpreted as lesions, thus distorting the specificity assessment. 10 datasets, excluded from successive analysis, were used for training purposes by unblinding the pathology’s results to the radiologist. This allowed their correct interpretation, and after repeating this process over 10 samples the radiologist felt more confident in routinely interpreting these features. Multi-planar reformation (MPR) in three planes was used in the assessment of the XPCI-CT images.

Histopathological analysis classified each tissue sample as belonging to one of the three above categories, and was used as the “gold standard” against which both XPCI-CT and conventional specimen imaging were compared. The tissue samples were orientated with inks prior to imaging, to allow images and histology to be viewed in the same orientation. The entire specimen was embedded, superior to inferior. Any lesion identified on imaging was located as a distance from superior aspect of the specimen, enabling the histology blocks to be levelled through to the correct distance. In all cases, a histological correlate of the lesion on imaging was identified. The other team members were kept blind to this classification.

2 × 2 contingency tables were created for image readings resulting from both conventional and XPCI-CT imaging (based on 99 and 101 specimen, respectively), to establish their sensitivity and specificity against tumour presence at margins through comparison against the histopathology gold standard. Where the presence of tumour at margins was classified as “uncertain” by the radiologist, this was counted as a “yes” in the contingency tables, based on the assumption that this would lead to the resection of additional tissue in a BCS procedure. Alternative tables excluding all uncertain calls are provided in the supplementary information. 95% Confidence Intervals (CIs) were obtained assuming the numbers correctly classified had a binomial distribution^37^.

## Supplementary Information

Supplementary Information.
