# Supplementary material for: Detection of involved margins in breast specimens with X-ray phase-contrast computed tomography
Source: Sci Rep. 2021 Feb 11;11:3663. doi: 10.1038/s41598-021-83330-w (PMC7878478; doi:10.1038/s41598-021-83330-w)
Supplement: Supplementary file 1 — Supplementary Information. [file 41598_2021_83330_MOESM1_ESM.docx]

**Detection of involved margins in breast specimens with x-ray phase-contrast computed tomography**

Lorenzo Massimi,*^a *^* Tamara Suaris,*^b^* Charlotte K. Hagen,*^a^* Marco Endrizzi,*^a^* Peter R. T. Munro,*^a^* Glafkos Havariyoun,*^a^* P. M. Sam Hawker,*^c^* Bennie Smit,*^c^* Alberto Astolfo,*^c^* Oliver J. Larkin,*^c^* Richard M. Waltham,*^c^* Zoheb Shah,*^d^* Stephen W. Duffy,*^d^* Rachel L. Nelan,*^d^* Anthony Peel,*^b^* J. Louise Jones,*^b,d^* Ian G. Haig,*^c^* David Bate,*^c^* and Alessandro Olivo*^a*^*

^a^ Department of Medical Physics and Biomedical Engineering, University College London, Gower St, London WC1E 6BT, UK

^b^ St Bartholomew’s Hospital, Barts Health NHS Trust, West Smithfields, London EC1A 7BE, UK

^c^ Nikon X-Tek Systems, Tring Business Centre, Icknield Way, Tring, Hertfordshire, HP23 4JX, UK

^d^ Barts and the London School of Medicine and Dentistry, Queen Mary University of London, Newark St, London E1 2AT, UK

*corresponding author a.olivo@ucl.ac.uk

**Supplementary Information**

**Exclusion of uncertain cases from the statistical analysis**

When analysing the main results of the study, we decided to include all cases where the radiologist was unsure of cancer presence at margins into the “yes” category, the underlying assumption being that in that case it would be safer to resect some additional tissue in the BCS procedure, and therefore that they would count as “yeses” for practical purposes. Here we present the tables that result from the exclusion of those cases.

Table ST1 focuses on the detection of lesions at the margin of the specimen by XPCI-CT and reveals a sensitivity of 82% (95% CI 67-91%) and a specificity of 88% (95% CI 75-95%). Table ST2 shows the same results for conventional specimen radiography, revealing a sensitivity of 33% (95% CI 20-49%) and a specificity of 87% (95% CI 75-94%).

|  | **XPCI-CT** | |  |
| --- | --- | --- | --- |
| **Pathology** | cancer | no cancer | Total |
| cancer | 37 | 8 | 45 |
| no cancer | 6 | 45 | 51 |
| Total | 43 | 53 | 96 |

***Table ST1****: 2 x 2 contingency table for cancer presence at margins as detected by XPCI-CT, in which all uncertain cases have been excluded.*

|  | **conventional specimen radiography** | |  |
| --- | --- | --- | --- |
| **Pathology** | cancer | no cancer | Total |
| cancer | 14 | 29 | 43 |
| no cancer | 7 | 48 | 55 |
| Total | 21 | 77 | 98 |

***Table ST2****: 2 x 2 contingency table for cancer presence at margins detected by specimen radiography, in which all uncertain cases have been excluded.*

As can be seen, in both cases the difference between the sensitivity and specificity values extracted while excluding uncertain cases or interpreting them as “cancer present” is extremely small, and well within the statistical uncertainty. It should however be noted that the overall fraction of excluded cases was very small in this study.

**Detection of tumour regardless of its position inside the specimens**

The main article reports the sensitivity and specificity analysis for the detection of cancer lesions reaching the edge of the specimen (margin involvement). Here we report on the same analysis for the detection of cancer lesions irrespective of whether these reach the specimen’s margins. Table ST3 shows the overall results for XPCI-CT in the detection of cancer lesions regardless of where they are located inside the specimen. This gives a sensitivity of 85% (95% CI 71-93%) and a specificity of 78% (95% CI 61-88%). Table ST4 reports the same results for conventional specimen imaging radiography. The sensitivity is 44%, 95% CI 29-60% and the specificity is 84%, 95% CI 72-92%.

|  | **XPCI-CT** | |  |
| --- | --- | --- | --- |
| **Pathology** | cancer | no cancer | Total |
| cancer | 40 | 7 | 47 |
| no cancer | 12 | 42 | 54 |
| Total | 52 | 49 | 101 |

***Table ST3****: 2 x 2 contingency table for cancer presence overall as detected by XPCI-CT*

|  | **conventional specimen radiography** | |  |
| --- | --- | --- | --- |
| **Pathology** | cancer | no cancer | Total |
| cancer | 19 | 24 | 43 |
| no cancer | 9 | 47 | 56 |
| Total | 28 | 71 | 99 |

***Table ST4****: 2 x 2 contingency table for cancer presence overall as detected by specimen radiography*

**2D (planar) imaging**

As well as CT images in minutes, the same scanner can be used to produce planar (2D) images in a few seconds. These can be “single-shot” or “multi-modal”. In “single shot” (25-26) or “hybrid” images, the conventional attenuation and phase effects coexist, with the former being enhanced by the latter (14,17,38). “Multi-modal” images simultaneously show separate attenuation, differential phase and “dark field” (or “ultra-small angle scatter”) information (16,39). An example is reported in Figure S1.


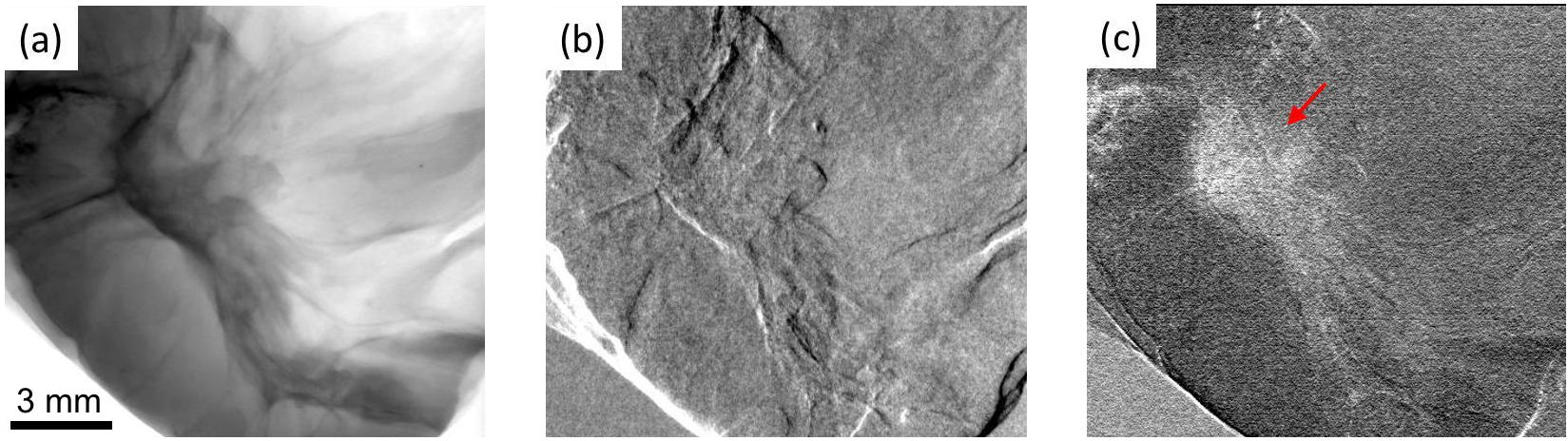


***Figure S1****: Multi-modal planar imaging options provided by the same scanner. (a) attenuation-based image, (b) differential phase image, (c) ultra-small angle scatter (“dark field”) image.*

The “dark field” image displays the presence of specimen inhomogeneities on a scale below the spatial resolution of the system. For example, the arrow in panel c indicates areas where the specimen is inhomogeneous below the system’s resolution. Dark field images of breast tissue have been proven to be potentially useful in detecting smaller calcified structures, including suggestions to use them to distinguish between benign and malignant calcifications (40).

**Fixed vs fresh breast tissue specimens**

At the beginning of the study we compared XPCI-CT slices of the same specimen before and after fixation in 10% formal saline, to confirm this would not significantly affect the outcomes. The two images were obtained in exactly in the same conditions, with the breadboard systems and the parameters listed in the “materials and methods” section of the main paper. Figure S2 shows example slices from the tissue both before and after fixation.


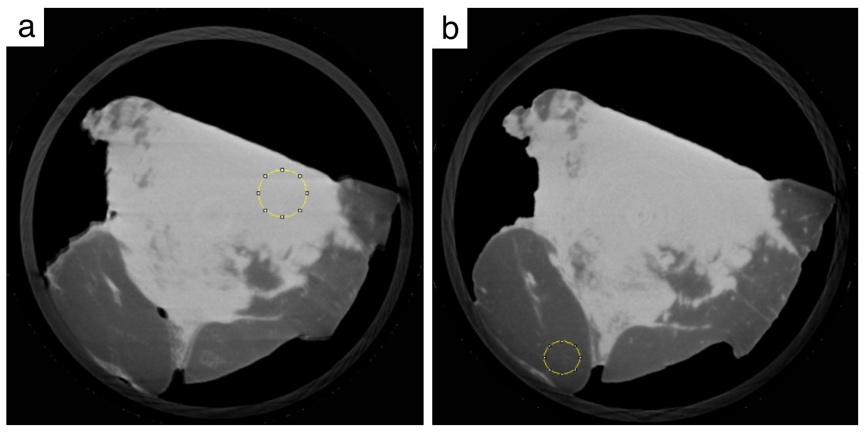


***Figure S2****: XPCI-CT images of the same breast tissue specimen before (a) and after (b) fixation in 10% formal saline.*

Despite the fresh tissue image suffering from a moderate motion artefact, visual inspection suggests contrast is not affected by formaldehyde fixation. To confirm this, regions of interest (ROIs) were selected in the same fibroglandular and fat regions of the specimen before and after fixation (these are indicted as yellow circles in panels a and b, respectively). Average voxel values were extracted from these same ROIs for both images, and are reported with their respective standard deviations in table ST5 below.

| Tissue type | Fibroglandular (V_fg_±σ_Vfg_) | Fat (V_f_±σ_Vf_) | Contrast (V_fg_ - V_f_)/V_fg_ |
| --- | --- | --- | --- |
| Fresh | 11,790±120 | 7,460±190 | 36.7% |
| Fixed | 11,770±140 | 7,380±180 | 37.3% |

***Table ST5****: average voxel content of fibroglandular and fat tissue, and fibroglandular vs fat image contrast before and after fixation in 10% formal saline.*

As can be seen from the table, not only is the variation in contrast negligible, but also the variation in extracted voxel values themselves are smaller than their natural variation inside the tissue, as expressed by their standard deviation.

**Additional References for Supplementary Information**

1. Olivo, A. et al. Low-dose phase contrast mammography with conventional x-ray sources. *Med. Phys.* **40**, 090701 (2013).
2. Endrizzi, M. et al. Hard x-ray dark field imaging with incoherent sample illumination. *Appl. Phys. Lett.* **104**, 024106 (2014).
3. Wang, Z. et al. Non-invasive classification of microcalcifications with phase-contrast x-ray mammography. *Nat. Commun.* **5**, 3797 (2014).
